# Twelve Weeks of Medium-Intensity Exercise Therapy Affects the Lipoprotein Profile of Multiple Sclerosis Patients

**DOI:** 10.3390/ijms19010193

**Published:** 2018-01-08

**Authors:** Winde Jorissen, Tim Vanmierlo, Inez Wens, Veerle Somers, Bart Van Wijmeersch, Jeroen F. Bogie, Alan T. Remaley, Bert O. Eijnde, Jerome J. A. Hendriks

**Affiliations:** 1 BIOMED, School of Life Sciences, Hasselt University, 3590 Diepenbeek, Belgium; winde.jorissen@uhasselt.be (W.J.); tim.vanmierlo@uhasselt.be (T.V.); veerle.somers@uhasselt.be (V.S.); jeroen.bogie@uhasselt.be (J.F.B.); 2 REVAL Rehabilitation Research Center, BIOMED Biomedical Research Institute, Faculty of Medicine and Life Sciences, Hasselt University, 3590 Diepenbeek, Belgium; inez.wens@uhasselt.be; 3 Revalidation and MS Center, 3900 Overpelt, Belgium; bart.vanwijmeersch@uhasselt.be; 4 Clinical Center, Department of Laboratory Medicine, National Institutes of Health Clinical Center (NIH), Bethesda, MD 20814, USA; Alan.Remaley@nih.gov

**Keywords:** multiple sclerosis, lipoproteins, cholesterol, training exercise

## Abstract

Multiple sclerosis (MS) is an inflammatory auto-immune disease of the central nervous system (CNS). Serum glucose alterations and impaired glucose tolerance (IGT) are reported in MS patients, and are commonly associated with the development of cardio-metabolic co-morbidities. We previously found that a subgroup of MS patients shows alterations in their lipoprotein profile that are similar to a pre-cardiovascular risk profile. In addition, we showed that a high-intensity exercise training has a positive effect on IGT in MS patients. In this study, we hypothesize that exercise training positively influences the lipoprotein profile of MS patients. To this end, we performed a pilot study and determined the lipoprotein profile before (controls, *n* = 40; MS patients, *n* = 41) and after (*n* = 41 MS only) 12 weeks of medium-intensity continuous training (MIT, *n* = 21, ~60% of VO_2max_) or high-intensity interval training (HIT, *n* = 20, ~100–200% of VO_2max_) using nuclear magnetic resonance spectroscopy (NMR). Twelve weeks of MIT reduced intermediate-density lipoprotein particle count ((nmol/L); −43.4%; *p* < 0.01), low-density lipoprotein cholesterol (LDL-c (mg/dL); −7.6%; *p* < 0.05) and VLDL size ((nm); −6.6%; *p* < 0.05), whereas HIT did not influence the lipoprotein profile. These results show that MIT partially normalizes lipoprotein alterations in MS patients. Future studies including larger patient and control groups should determine whether MIT can reverse other lipoprotein levels and function and if these alterations are related to MS disease progression and the development of co-morbidities.

## 1. Introduction

Multiple sclerosis (MS) patients are reported to suffer from an increased serum glucose concentration, impaired glucose tolerance (IGT) [1], higher insulin resistance (IR) [2,3] and hyperinsulinemia [4], all of which are cardiometabolic risk factors. Importantly, vascular comorbidity is associated with more rapid disability progression in multiple sclerosis [5]. Moreover, several studies suggest an association between the lipoprotein profile of MS patients and MS disease processes such as lesion formation, blood-brain-barrier function, disability, and MRI outcome [6,7,8,9,10,11]. In literature, the findings with regard to possible alterations of the lipoprotein profile in MS patients are inconclusive [6,7,8,9,12,13,14,15,16,17,18,19,20,21]. Interestingly, we and others recently demonstrated a pre-cardiovascular-like risk lipoprotein profile in a subgroup of MS patients as evidenced by smaller high density lipoprotein (HDL) and low density lipoprotein (LDL) particles, increased triglycerides (TG), very low density lipoprotein associated TGs (VLDL-TG) and VLDL size, and a higher lipoprotein insulin resistance index (LP-IR) [3,13]. Changes in both total and LDL but not VLDL cholesterol were previously described to be significantly correlated to the mean number of enhancing lesions over time [9]. Importantly, pre-cardiovascular-like risk lipoprotein profile factors can be affected by exercise. At present, exercise and rehabilitation therapy are used to remediate disability and the overall functional capacity of MS patients [22,23,24,25]. Substantially improved exercise capacity and muscle strength have been reported following various modes (duration, intensity) of exercise.

During the last decade exercise therapy studies [26], showed that medium-intensity continuous training (MIT) exercise induces low to moderate (+10–15%) improvements in muscle strength and exercise capacity in MS patients. High-intensity exercise training (HIT) programs in MS patients and in a rat experimental autoimmune encephalomyelitis (EAE) model for MS have shown to further improve muscle strength and exercise capacity (+25–60%) and have indicated that HIT regimens are safe, well-tolerated, and render the highest functional benefits [25,27].

Because MS-related inactivity also affects several cardiometabolic risk factors such as blood lipid profiles it is important to not only investigate the impact of MIT or HIT on functional parameters (e.g., muscle strength and exercise capacity) but also to measure their effect on health-related measures such as lipoprotein levels. With respect to the latter it is important to note that in sedentary subjects and in type II diabetes patients it was recently shown that MIT programs improves whole body insulin action, glycemic control and plasma lipids substantially more than vigorous exercise [28,29].

As the optimal training duration, frequency and intensity, and the underlying mechanisms of the impact of training on MS are not fully clear yet, we aimed to investigate in this pilot study the effect of two different training regimens (e.g., a 12 week MIT and HIT exercise/rehabilitation program) on blood lipoprotein profiles of MS patients. Based on the above findings, we hypothesized that both training programs affect/normalize the lipoprotein profile of MS patients with a more pronounced effect after MIT. Our results indicate that MIT affects/normalizes lipoprotein alterations in MS patients. Further studies in larger patient cohorts are needed to address whether MIT can influence these alterations after taking into account the possible contribution of daily habits, treatment and the different MS types. Such studies will determine whether a MIT exercise regimen can influence MS disease progression and the development of co-morbidities.

## 2. Results

### Twelve Weeks of Medium-Intensity Endurance Training Affects the Lipoprotein Profile of MS Patients

To determine which changes in lipoprotein levels occurs in MS patients before and after training, we analyzed a standardized set of lipoprotein parameters with the Vantera Clinical Analyzer^®^ [30]. At baseline, the different lipoproteins and their subclasses, as well as a lipoprotein-based insulin resistance index (LP-IR) were determined in MS patients and healthy controls using NMR. MS patients showed significantly smaller LDL particles (LDL size (nm), *p* < 0.05), an increase in the amount of large VLDL particles (Large VLDL particle count (nmol/L), *p* < 0.001) and a higher lipoprotein insulin resistance index (LP-IR, *p* < 0.001) compared to controls (Table 1). MS patients were randomly assigned to the MIT (*n* = 21) or HIT (*n* = 20) group based on age, gender and body mass index (BMI). The groups respectively performed a medium-intensity endurance training (MIT) or a high-intensity interval training (HIT) program for 12 weeks.

After 12 weeks, the lipoprotein profile of the MS patients was again measured using NMR. Due to differences between the MIT and HIT groups at baseline we did not analyze differences POST-training between the two different training groups. After 12 weeks, the medium-intensity training program reduced low density lipoprotein-cholesterol (LDL-c) levels, VLDL size and intermediate density lipoprotein (IDL) particle count. The high-intensity training program had no effect on the measured lipoprotein profile parameters (Table 2).

**Figure 1:**
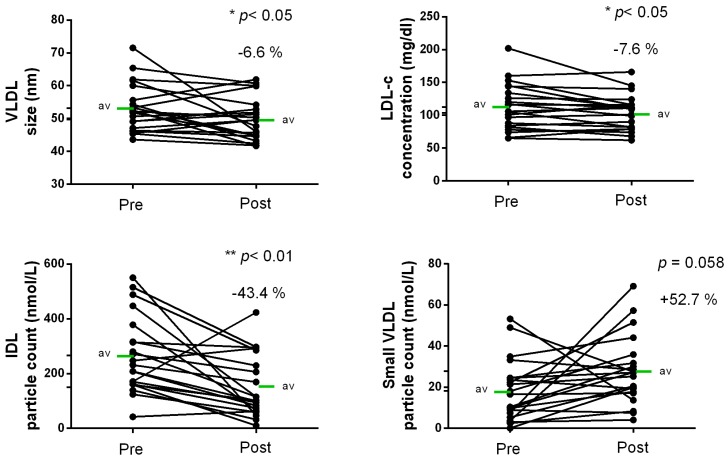
Graphical display of the significant results of Table 1. Twelve weeks of MIT ameliorates the lipoprotein profile of multiple sclerosis (MS) patients. Shown are individual observations, measured by NMR pre and post 12 weeks of medium-intensity training (MIT). * *p* < 0.05, ** *p* < 0.01.

MIT showed a trend towards an increase in small VLDL particle count (+52.7%; *p* < 0.058) and displayed a significant reduction in IDL particle count (−43.4%; *p* < 0.01), LDL cholesterol (LDL-c (mg/dL); −7.6%; *p* < 0.05) and VLDL size (−6.6%; *p* < 0.05) after the 12-week training program (Table 1 and Figure 1).

## 3. Discussion

The aim of this study was to determine the effect of a medium-intensity endurance training (MIT) and a high-intensity interval training (HIT) program on lipoprotein alterations in MS patients. At baseline, MS patients showed a higher LP-IR, accompanied by smaller LDL and larger VLDL particles and a trend towards a reduction in HDL size (which is likely due to a reduction in the amount of large HDL particles) and an increased VLDL-TG content. 12 weeks of MIT influenced several lipoprotein parameters but had no significant effect on LP-IR; LDL size or large VLDL particle count. The 12-week HIT program had no significant influence on any of the measured lipoprotein profile parameters. It should be noted that the results from this pilot study should be confirmed in larger study cohorts of MS patients.

In non-MS subjects, atherogenic lipoprotein alterations as well as impaired glucose tolerance (IGT), and insulin resistance (IR) are important precursors for a wide variety of cardiovascular diseases. These parameters increase the overall cardiovascular disease (CVD) risk via mechanisms that affect the endothelium, the vascular wall, and smooth muscle cells [31,32,33].

The observed alterations of the lipoprotein profile of MS patients at baseline are characteristic of a “pre”-cardiovascular-like risk lipoprotein profile [34], and confirm our previous observations in another MS patient cohort [3]. In the previous study, we showed that relapsing-remitting MS (RRMS) patients display smaller LDL and a consequent decrease in large LDL compared to healthy subjects and progressive MS patients. In addition, RRMS patients with a low BMI displayed an even more pronounced altered lipoprotein profile with an increase in small HDL, LP-IR, TG, VLDL-TG, and VLDL size. The findings from the current study are in line with the observations in low BMI RRMS subjects in our previous study. However, we did not observe an increase in small HDL. Due to the fact that the patient group tested was relatively small, we were not able to test any confounding effects of BMI, gender, age and type of MS in this study.

Treatment of CVD risk precursors in non-MS subjects includes pharmacological interventions and lifestyle modifications [35,36,37,38]. One of the standard treatments for increased cardiometabolic risk in non-MS subjects involves increased “continuous” medium-intensity physical exercise applied during longer duration exercise training sessions (i.e., a high amount of time spent on exercise training) [39]. Recent studies, however, suggest that short duration (i.e., short exercise sessions) HIT can also yield a broad range of physiological gains [40].

Our results show that 12 weeks of MIT, but not HIT, has beneficial effects on lipoprotein alterations associated with a CVD risk profile in MS patients. More specific, after 12 weeks of MIT, MS patients displayed lower levels of IDL particle count, LDL-cholesterol, and had a reduction in the size of VLDL particles with a trend towards increased small VDL.

IDL is processed from VLDL particles, and IDL is usually very low or absent in fasting blood from healthy subjects. IDL can trigger the growth of atherosclerotic plaques [41,42]. Also, IDL increases the chance of developing soft plaques in carotid arteries, which easily fragment, release debris, and can cause stroke [43,44]. Thus, the reduction in IDL particle count which was observed after 12 weeks of MIT can be considered as a positive effect on the lipoprotein profile of MS patients.

Although there has been a lot of debate about the role of LDL-c in the development of CVD, several longitudinal studies have identified LDL-c as a risk factor for CVD incidence, reoccurrence and fatal outcome [45]. Moreover, previous studies show a relationship between LDL-c reduction and the reduction of cardiovascular risk, regardless of initial LDL-c levels or whether patients achieved “target” LDL-c values of <100 mg/dL [46]. Despite the absence of a difference for LDL-c between MS patients and healthy controls at baseline, the decrease in LDL-c has a beneficial influence on the lipoprotein profile of MS patients. The clinical relevance of reductions in LDL-c levels is highlighted by a recent review of Zhornitsky and colleagues which summarizes significant associations between higher LDL-c levels and increased EDSS scores, new and enlarging T2 lesions and increased grey matter atrophy in MS patients [47].

The increase in large VLDL particle count, which was observed in MS patients at baseline, is typically associated with the presence of insulin resistance. These large, TG-rich VLDL particles are considered a major contributing factor to an atherogenic dyslipidemic lipoprotein profile [48,49] and are precursors of small, dense LDL particles [50]. In line with this, we observed a decrease in LDL-size in MS patients at baseline. Smaller, denser LDL particles are considered to be more atherogenic because of a decreased capacity to bind to the LDL receptor, which leads to an increased residence time in the plasma [51] and an increased susceptibility to oxidation [52].

The observed positive effect of MIT on alterations of the lipoprotein profile of MS patients in this study, is in line with previous reports showing that exercise volume is more important than exercise intensity to influence lipoprotein parameters in the blood [53]. This may explain why we observed beneficial effects on the lipoprotein profile of MS patients performing MIT and not HIT.

Although the exact mechanisms underlying the effect of exercise on the lipoprotein profile are unclear, exercise is known to enhance the ability of skeletal muscles to utilize lipids as opposed to glycogen, hereby reducing plasma lipid levels [54]. Importantly, endurance training primarily involves the aerobic energy pathway, in which fatty acids will be used as an energy substrate for the Krebs cycle following approximately 20 min of sustained activity. In contrast, HIT initially results in decreased adenosine triphosphate (ATP) stores followed by decreased glycogen stores [55] through anaerobic glycolysis [56]. Nonetheless, a training study of O’Donovan and colleagues in healthy subjects, indicates a higher effectiveness of HIT on lipoprotein alterations, after a training program of 24 weeks [57]. Taken together, the above studies indicate that both the MIT and HIT training programs may affect cardiometabolic risk lipoprotein alterations in MS patients, albeit after different timeframes. All of the identified alterations can be seen as (interrelated) individual contributors to a panel of CVD-risk estimation parameters as a whole, which together help predicting the risk for CVD in addition to the formerly used HDL-c levels. Importantly, multiple aspects beyond training may have influenced the baseline and post-training levels as dietary habits, smoking habits, possible underlying inflammatory processes in MS patients, etc. may readily influence their lipoprotein profile.

This study confirms our previous findings demonstrating that lipoprotein levels and size are altered in MS and shows that MIT can ameliorate these changes. However, it should be taken in to account that the small sample size of our study did not allow us to define the possible confounding effects of therapy, age, gender, BMI, MS disease type, etc. In addition, we do not have any information on disease onset and we were not able to directly compare the MIT with the HIT group as these groups already differed significantly on their lipoprotein profile at baseline. It would be interesting to follow up these patients in time to determine intrapatient changes in lipoprotein levels, but also to determine correlations of these changes with clinical outcome parameters such as the number of new Gd + T1 lesions, active T2 lesions and combined unique active (CUA) lesions.

In summary, our results suggest that a 12-week medium-intensity cardiovascular exercise/rehabilitation program can improve blood cholesterol profiles in MS patients. These results suggest that exercise/rehabilitation therapy in MS patients may positively modify MS disease progression and may reduce the development of co-morbidities such as CVD. More research using larger study cohorts, taking into account possible effects of the different types of MS as well as of daily habits is needed to evaluate the effect of training programs on the lipoprotein profile of MS patient subsets.

## 4. Materials and Methods

### 4.1. Subjects

A total of 40 healthy controls and 41 MS patients were recruited after providing detailed information on all the experimental procedures and informed written consent. The study was approved on 11 June 2012; amendment approved on 22 April 2014 by the Medical Ethics Committees of Hasselt University and UZ Leuven (S54401 Ref. ML8336). MS patients were included independent of their MS-related medication status. Patients were either untreated or treated with interferon β (Rebif^®^), glatiramer acetate (Copaxone^®^), fingolimod (Gilenya^®^), natalizumab (Tysabri^®^), or Alemtuzumab (Campath^®^). An overview of the different therapies in the patient subgroups is shown in the Supplemental Table S1. Exclusion criteria for patients and controls were reported hypercholesterolemia, cardiovascular diseases, diabetes, pregnancy and treatment with glucose cholesterol modifying agents. Supplemental Table S2 shows the distribution of the different MS disease types included in this study.

### 4.2. Study Design

The patients were recruited via the MS clinic in Overpelt (Belgium), flyers in hospitals, information evenings, and a participation list from previous studies at our institute. Following study inclusion, baseline serum samples of MS patients and healthy controls were collected and analyzed using NMR. The healthy control group was only used for baseline comparisons and was not randomized or involved in the training program as its sole purpose was the identification of cardiovascular risk parameters in the lipoprotein profile of the MS study population. After baseline measurements, the MS subjects were randomized via random sampling allocation using SPSS into 2 exercise intervention groups (12 weeks) matching for age, gender and BMI. Training interventions involved medium-intensity cardiovascular training (MIT, *n* = 21, ~60% of VO_2max_) or high-intensity interval training (HIT, *n* = 20, ~100–120% of VO_2max_). MIT (5 sessions/2-week cycle, 48 h recovery between sessions) involved cardiovascular (walking/running, 1 × 6 min/session→2 × 10 min/session Technogym^®^) and resistance (leg press, leg curl, leg extension, vertical traction, arm curl and chest press, 1 × 10 repetitions→2 × 20 repetitions @ W_max_, Technogym^®^) training. To train at similar relative workloads leg resistance training was performed unilaterally [58]. When required, continuous and interval training intensity were adapted based on individual disability levels and capabilities. HIT intervention (5 sessions/2-week cycle, 48 h recovery between sessions) also involved a cardiovascular and resistance training part. Cardiovascular training involved a 5 min warm up (cycle ergometer) and five high-intensity interval cycle bouts. During the first 6 weeks, exercise duration increased from 5 × 1 min to 5 × 2 min maximal exercise (HR @ 100% VO_2max_, 1 min rest intervals). During the final 6 weeks of training, intensity of the exercise interval bouts (5 × 2 min) increased to heart rates corresponding to 100–120% VO_2max_. During HIT resistance training was similar to MIT. VO_2max_ was measured using breath-by-breath gas analysis (ergospirometry). There were no drop-out rates during the training program. Following 12 weeks of MIT or HIT post intervention serum samples were obtained and analyzed similar to baseline.

### 4.3. Sample Processing

Serum was obtained after centrifugation of clotted fasting blood samples at 400 g for ten minutes, and was immediately stored at −80 °C by the University Biobank Limburg (UBiLim). Study information on healthy controls (age, gender, weight, and length) and MS patients (age, gender, weight, length, therapy, and EDSS score) was obtained from UBiLim.

### 4.4. Nuclear Magnetic Resonance Spectroscopy

Fasting serum samples were thawed and 500 μL of sample was immediately analyzed using the 400-MHz proton Vantera Clinical Analyzer^®^ (Liposcience, Raleigh, NC, USA) [30], the first NMR providing lipoprotein tests approved by the US Food and Drug Administration for use as a clinical instrument source. Particle size, concentration and subclass (large, medium, small) distribution were measured for all lipoprotein classes (HDL, LDL, IDL, VLDL). In addition, TG concentrations and VLDL-TG were measured. The NMR analysis involved measurement of the sample, deconvolution of the signal, and conversion of the signal into specific lipoprotein subclass concentrations. The 400-MHz proton NMR spectrum of each serum sample was measured, producing a signal at ~0.8 ppm, which was derived from the methyl group protons of the lipids carried in the lipoprotein subclasses. The signals at ~0.8 ppm had unique and distinctive frequencies and line shapes, each of which were accounted for in the deconvolution analysis model. The lipoprotein insulin resistance (LP-IR) index measured by the NMR is calculated from three lipoprotein subclasses (large VLDL, small LDL and large HDL) and three particle sizes (VLDL, LDL and HDL). The LP-IR is strongly associated with the homeostasis model assessment of insulin resistance (*r* = 0.51) and glucose disposal rates (*r* = −0.53) [59], the former being more reflective of hepatic and the latter of peripheral insulin sensitivity. The results are reported on a scale ranging from 0 (most insulin sensitive) to 100 (most insulin resistant).

### 4.5. Statistical Analysis

For descriptive statistics, two-tailed unpaired *t*-tests or Mann-Whitney tests were performed in GraphPad Prism (Windows version 6). Differences in gender were assessed using chi-square tests. For pre-post delta effects (i.e., the change “pre” versus “post” training) within the different training groups, two-tailed paired *t*-tests or Wilcoxon tests were performed. Statistical significance is presented as * *p* < 0.05, ** *p* < 0.01, and *** *p* < 0.001 in all tables and figures. Data are presented as mean ± SEM.

## Figures and Tables

**Table 1:** Descriptive statistics and lipoprotein profile measurements for the study population at baseline.

Parameter	HC	MS	*p* Value
*N*	40	41	
Age	47.5 ± 1.8	46 ± 1.5	0.511
Male gender, %	12 (30%)	19 (46%)	0.17
BMI	24.3 ± 0.5	24.7 ± 0.7	0.919
EDSS score	NA	2.8 ± 0.4	NA
Conventional lipid panel			
Total cholesterol, mg/dL	191.6 ± 5.7	181.4 ± 5.1	0.185
Triglycerides, mg/dL	97.8 ± 7.1	105.3 ± 5.0	0.104
HDL-c, mg/dL	66.6 ± 1.8	62.0 ± 2.9	0.175
LDL-c, mg/dL	115.3 ± 5.6	110.3 ± 5.0	0.501
Lipoprotein subclasses			
HDL			
Size nm	9.7 ± 0.1	9.4 ± 0.1	0.075
Total particle count, μmol/L	36.4 ± 0.8	35.3 ± 1.0	0.413
Small particle count, μmol/L	13.0 ± 1.0	14.3 ± 1.1	0.368
Medium particle count, μmol/L	13.2 ± 1.0	12.8 ± 1.3	0.758
Large particle count, μmol/L	8.6 ± 0.5	7.4 ± 0.7	0.084
LDL			
Size, nm	21.4 ± 0.1	21.1 ± 0.1 *	**0.033**
Total particle count, nmol/L	1122.4 ± 63.4	1106.6 ± 52.3	0.931
Small particle count, nmol/L	280.4 ± 38.9	286.0 ± 36.1	0.794
Large particle count, nmol/L	535.2 ± 32.4	506.2 ± 33.2	0.535
IDL			
Particle count, nmol/L	192.8 ± 19.3	204.5 ± 20.9	0.794
VLDL			
Size, nm	47.9 ± 1.0	50.7 ± 1.1	0.115
Total particle count, nmol/L	40.8 ± 4.1	43.2 ± 3.6	0.663
Small particle count, nmol/L	21.7 ± 2.0	24.1 ± 2.6	0.470
Medium particle count, nmol/L	17.2 ± 2.6	16.1 ± 2.4	0.882
Large particle count, nmol/L	2.9 ± 0.5	4.0 ± 0.4 ***	**0.0004**
VLDL-Triglycerides, mg/dL	59.7 ± 5.4	67.7 ± 4.1	0.085
LP-IR index (0–100)	29.7 ± 2.7	44.3 ± 2.8 ***	**0.0003**

Table 1 provides an overview of characteristics of the study population and of their lipid and lipoprotein profile at baseline. Healthy control (HC), multiple sclerosis (MS), body mass index (BMI), high denity lipoprotein (HDL), low density lipoprotein (LDL), intermediate density lipoprotein (IDL), very low density lipoprotein (VLDL).Values are means ± SEM. Significant observations in bold. * versus HC; * *p* < 0.05, *** *p* < 0.001; NA: not applicable.

**Table 2:** Pre and post training descriptive statistics and lipoprotein profile measurements in the multiple sclerosis (MS) study population.

Parameter	Medium-Intensity Training			High-Intensity Training		
*N*	21			20		
Age	48.0 ± 2.7			44.0 ± 2.6		
Male gender, %	10 (47.6%)			9 (45%)		
BMI	24.6 ± 1.3			24.7 ± 1.0		
EDSS score	3.2 ± 0.4			2.52 ± 0.3		
	**PRE**	**POST**	***p* value**	**PRE**	**POST**	***p* value**
Conventional lipid panel						
Total cholesterol, mg/dL	180.7 ± 7.6	176.7 ± 7.6 (−2.2%)	0.42	182.1 ± 6.9	183.0 ± 7.7 (+0.5%)	0.43
Triglycerides, mg/dL	106.2 ± 8.5	106.9 ± 6.6 (+0.7%)	0.90	104.3 ± 5.4	116.0 ± 9.4 (+11.2%)	0.33
HDL-c, mg/dL	60.4 ± 4.2	61.2 ± 4.7 (+1.3%)	0.60	63.6 ± 4.0	64.2 ± 4.0 (+0.9%)	0.75
LDL-c, mg/dL	112.5 ± 7.7	103.9 ± 5.8 ^$^ (−7.6%)	**0.042**	107.9 ± 6.5	106.9 ± 8.3 (−0.9%)	0.14
Lipoprotein subclasses						
HDL						
Size nm	9.4 ± 0.2	9.4 ± 0.1 (no % change)	0.58	9.5 ± 0.1	9.5 ± 0.1 (no % change)	0.55
Total particle count, μmol/L	34.6 ± 1.5	35.08 ± 1.5 (+1.4%)	0.63	36.1 ± 1.3	36.7 ± 1.2 (+1.7%)	0.31
Small particle count, μmol/L	15.1 ± 1.5	14.0 ± 1.8 (−7.3%)	0.41	13.5 ± 1.6	12.7 ± 1.6 (−5.9%)	0.64
Medium particle count, μmol/L	11.7 ± 1.8	12.6 ± 2.0 (+7.7%)	0.48	14.0 ± 2.0	15.5 ± 1.7 (+10.7%)	0.92
Large particle count, μmol/L	6.6 ± 0.9	7.2 ± 1.0 (+9.1%)	0.11	8.2 ± 0.9	8.4 ± 0.9 (+2.4%)	0.80
LDL						
Size, nm	21.1 ± 0.1	21.2 ± 0.1 (+0.5%)	0.60	21.1 ± 0.1	21.0 ± 0.1 (−0.5%)	0.06
Total particle count, nmol/L	1108.1 ± 79.1	1044.2 ± 68.1 (−5.8%)	0.10	1105.0 ± 70.1	1114.3 ± 101.2 (+0.8%)	0.33
Small particle count, nmol/L	233.1 ± 44.9	279.4 ± 53.1 (+19.9%)	0.21	341.6 ± 55.6	348.4 ± 72.5 (+2.0%)	0.86
Large particle count, nmol/L	497.7 ± 49.3	514.5 ± 40.1 (+3.4%)	0.64	515.2 ± 45.3	504.9 ± 51.1 (−2.0%)	0.79
IDL						
Particle count, nmol/L	265.6 ± 30.4 ^††^	150.3 ± 24.5 ^$$^ (−43.4%)	**0.002**	140.4 ± 21.0	156.1 ± 24.5 (+11.2%)	0.74
VLDL						
Size, nm	53.2 ± 1.6 ^†^	49.7 ± 1.4 ^$^ (−6.6%)	**0.047**	48.0 ± 1.1	49.4 ± 1.1 (+2.9%)	0.09
Total particle count, nmol/L	39.1 ± 5.6	47.0 ± 5.0 (+20.2%)	0.10	47.5 ± 4.3	50.3 ± 6.3 (+5.9%)	0.65
Small particle count, nmol/L	18.2 ± 3.2 ^†^	27.8 ± 3.6 (+52.7%)	0.058	30.3 ± 3.8	29.9 ± 3.6 (−1.3%)	0.88
Medium particle count, nmol/L	17.8 ± 4.2	15.6 ± 2.0 (−12.4%)	0.98	14.3 ± 2.3	16.8 ± 4.1 (+17.5%)	0.84
Large particle count, nmol/L	4.4 ± 0.7	4.4 ± 0.6 (no % change)	0.79	3.5 ± 0.3	4.5 ± 0.7 (+28.6%)	0.08
VLDL-Triglycerides, mg/dL	68.7 ± 7.2	71.7 ± 5.1 (+4.4%)	0.71	66.5 ± 4.2	77.4 ± 8.0 (+16.8%)	0.28
LP-IR index (0–100)	48.8 ± 4.4	43.3 ± 3.7 (−11.3%)	0.09	39.6 ± 3.4	43.0 ± 4.6 (+8.6%)	0.15

Table 2 shows pre- and post- training measurements for the MIT and HIT MS groups. Values are means ± SEM. Significant observations in bold. ^$^: versus pre-training; ^$^ *p* < 0.05, ^$$^ *p* < 0.01; ^†^: versus HIT; ^†^ *p* < 0.05, ^††^ *p* < 0.01.

## Supplementary Materials

Supplementary materials can be found at www.mdpi.com/1422-0067/19/1/193/s1.
