# Supplementary material for: Twelve Weeks of Medium-Intensity Exercise Therapy Affects the Lipoprotein Profile of Multiple Sclerosis Patients
Source: Int J Mol Sci. 2018 Jan 8;19(1):193. doi: 10.3390/ijms19010193 (PMC5796142; doi:10.3390/ijms19010193)
Supplement: Supplementary file 1 [file ijms-19-00193-s001.pdf]

Supplemental Table S1: MS patient therapies

	**RRMS (*n*)**	**Progressive MS (*n*)**
No treatment	5	2
Interferon β (Rebif®)	16	8
Glatiramer acetate (Copaxone®)	1	2
Fingolimod (Gilenya®)	4	1
Natalizumab (Tysabri®)	7	2
Alemtuzumab (Campath®)	1	2

Supplemental Table S2: MS patient disease types per training group

	**MIT (*n*=21)**		**HIT (*n*=20)**	
MS TYPE	MALE (n)	FEMALE (n)	MALE (n)	FEMALE (n)
RRMS	4	6	7	9
SPMS	5	5	2	2
PPMS	1	0	0	0
